# *Salvia miltiorrhiza*: insights on the protective effect and mechanism of myocardial ischemia-reperfusion injury

**DOI:** 10.1590/1414-431X2025e14723

**Published:** 2025-10-06

**Authors:** Qi Lan, Li Chen, Ming-Tai Chen, Zhen-Xun Wan, Ting Peng, Maryam Mazhar, Ping Liu, Gang Luo, Yan Jiang, Meng-Nan Liu

**Affiliations:** 1 Affiliated Traditional Chinese Medicine Hospital, Southwest Medical University, Luzhou, Sichuan, China; 2 Department of Cardiovascular Disease, Shenzhen Traditional Chinese Medicine Hospital, Shenzhen, Guangdong, China

**Keywords:** Salvia miltiorrhiza, Active ingredients, Myocardial ischemia-reperfusion injury, Pathological mechanism, Mechanism of action

## Abstract

Myocardial ischemia-reperfusion injury (MIRI), a common secondary complication of cardiovascular diseases (CVDs), leads to significant psychological and physiological distress in patients. Pathophysiological reactions including inflammatory response, oxidative stress injury, platelet aggregation, vascular endothelial dysfunction, and programmed cell death are involved in the pathogenesis of MIRI. Prolonged use of conventional therapies (e.g., NSAIDs, calcium channel blockers, beta-blockers, and antiplatelet agents) may exacerbate cardiovascular damage due to adverse effects. Thus, identifying complementary and alternative therapies with better efficacy and safety profile is imperative. Unlike single-target pharmacological approaches, *Salvia miltiorrhiza* Bunge exhibits pleiotropic effects by modulating multiple pathways, including inflammation, oxidative stress, and vascular function. This review summarizes the protective mechanisms of *Salvia miltiorrhiza* against MIRI, highlighting its potential as a translational therapy for MIRI and guiding future preclinical studies.

## Introduction

Cardiovascular diseases (CVDs) account for one-third of the total mortality rate around the globe (1). Acute myocardial infarction (AMI) is the most severe of CVDs with more than 3 million acute ST-segment elevation myocardial infarctions (STEMI) occurring every year (2). The first-line treatment of AMI patients is reperfusion therapy, but untimely reperfusion therapy may be accompanied by a series of pathophysiological responses including inflammatory reaction, peroxidation, platelet aggregation, and endothelial dysfunction, which aggravates the death of cardiomyocytes and leads to myocardial injury, referred to as myocardial ischemia-reperfusion injury (MIRI) (3).

The dried roots of *Salvia miltiorrhiza* Bunge (*Salvia miltiorrhiza*) have been widely used in China to treat CVDs, such as coronary heart disease, myocardial infarction, angina pectoris, and atherosclerosis. Numerous studies have proven that more than 30 lipophilic compounds with diterpene quinone structure (tanshinone I-VI, tanshinol A, etc.) and more than 50 hydrophilic compounds with phenolic acid structure (danshensu, tanshinone A, tanshinone B, etc.) in the chemical constituents of *Salvia miltiorrhiza* are effective in preventing and treating CVDs (4). Studies have shown that *Salvia miltiorrhiza* has several effects, including anti-inflammatory, inhibition of oxidative stress, regulation of platelet aggregation, and programmed cell death in the subsequent treatment of MIRI (5). However, there is currently no systematic study summarizing the mechanism of *Salvia miltiorrhiza*'s effect on MIRI. Moreover, studies have focused on *in vitro* and animal models and lack support from large-scale, high-quality clinical trials. Therefore, future research should aim to systematically elucidate the protective effect and mechanism of *Salvia miltiorrhiza* through high-quality clinical trials and deeper mechanism exploration, in order to provide more effective strategies for the treatment of MIRI.

This review aims to clarify the pathophysiological mechanisms of MIRI and discuss the effects of the active ingredients of *Salvia miltiorrhiza* on the pathogenesis of MIRI through various pathways and targets.

### Key compounds of *Salvia miltiorrhiza*: structure, pharmacokinetics, and proprietary Chinese medicines

*Salvia miltiorrhiza*, which belongs to the Salvia genus of Labiatae, is known for its medicinal properties. Its dried roots and rhizomes are widely used as one of the earliest traditional Chinese medicines. Pharmacological studies have found that CVDs are prevented and treated by the effects of *Salvia miltiorrhiza* by dilating coronary arteries, regulating blood lipids, improving microcirculation, reducing blood pressure, and combating thrombosis. Given that the complexity of the active components and the diversity of potential targets of *Salvia miltiorrhiza*, the bioactive components of *Salvia miltiorrhiza* were analyzed using the Traditional Chinese Medicine Systems Pharmacology Database and Analysis Platform (TCMSP, https://old.tcmsp-e.com/tcmsp.php). The TCMSP database is a unique systemic pharmacology platform for herbal medicines that captures the relationship between drugs, targets, and diseases (6). Based on screening criteria of oral bioavailability (OB) and drug-like (DL) properties of ≥30% and ≥0.18, 65 compounds were screened by literature review and manual screening, then 11 pharmacologically significant compounds of *Salvia miltiorrhiza* were selected. Structure, molecular formula, molecular weight, and isomeric/canonical SMILES (Simplified Molecular Input Line Entry System) were obtained from PubChem (https://pubchem.ncbi.nlm.nih.gov) as shown in Supplementary Table S1.

### Compounds specific to *Salvia miltiorrhiza* that have been widely studied

According to the dissolution characteristics of *Salvia miltiorrhiza*, its common compounds are divided into four classes: i) water-soluble phenolic acids (salvianolic acid A and salvianolic acid B); ii) lipid soluble diterpenoid quinones (tanshinone I, tanshinone IIa, tanshinone IIb, miltirone, tanshinol B, cryptotanshinone, and sclareol); iii) triterpenoid (α-amyrin), and iv) flavonoid (luteolin). Salvianolic acid B usually accounts for more than 50% of all the composition and is highly water-soluble. There is some evidence that salvianolic acid B has extremely low OB in rats because of its instability in the gastrointestinal tract, absorption by gastrointestinal epithelial cells but at a non-specific site, and then metabolism in the liver (7). The maximum plasma concentration (Cmax) of salvianolic acid A was observed to have a linear increase in the stomach of rats, the majority of which was excreted via feces, followed by bile and urine (8). Tanshinone I, present in traditional decoctions, was found to possess a broad first-pass effect with minimal absorption in the human body. However, granular powder formulations revealed extreme variations in Cmax, in time of Cmax occurrence (Tmax), and in elimination half-life (t1/2) (5). The Cmax and t1/2 values of tanshinone IIa in the human body after traditional decoction administration were lower than those of granular powder preparations (9). Tanshinone in the traditional decoction appeared rapidly in human plasma with a longer half-life period, which is consistent with the results in rats and rabbits (10). The OB of tanshinone IIb in rats was low and its Cmax, Tmax, and area under the curve (AUC) increased to a small degree with ascending dose (6). The low bioavailability of miltirone in rats may be related to its poor intestinal epithelial cell membrane permeability and hepatic first-pass effect, as well as to its metabolites in rat plasma and wide tissue distribution (11). In contrast, tanshinol B has excellent intestinal absorption rate, skin permeability, and high Caco-2 permeability (12). The bioavailability and t1/2 of cryptotanshinone were higher in dogs than in rats. In rats, the highest accumulation of cryptotanshinone was observed in the intestine, lungs, and liver after oral administration and in the lungs, liver, and heart after intravenous administration (5). After intravenous administration of sclareol to rats, it was deposited in the tissues and cleared slowly from the body (13). The OB and gastrointestinal absorption of α-amyrin, calculated by SwissADME, were found to be low (14). Luteolin, a flavonoid commonly found in a variety of medicinal plants, has been widely studied and applied due to its multiple biological activities and pharmacological properties (15). Due to the poor solubility of luteolin, coupled with its high first elimination rate and slow intestinal absorption, some studies have found that the OB of luteolin in rats is the lowest based on the principle that the bioavailability of luteolin varies with the administration route (16). Luteolin and its metabolites are potent and most of them are excreted in urine and bile within 24 h in rats (15).

### Proprietary Chinese medicines related to *Salvia miltiorrhiza*

In China, a large number of proprietary Chinese medicines containing *Salvia miltiorrhiza* as the main ingredient are widely used in clinics (as shown in Supplementary Table S2). Currently, several *Salvia miltiorrhiza* compound preparations have been validated for their clinical efficacy, such as Fufang Danshen Dripping pills, Compound Danshen tablets, Guanxinning tablets, and Jiawei Danshen decoction. Cardiac function and morphology after AMI were improved by Compound Danshen Dripping pills, which contains *Salvia miltiorrhiza*, *Panax notoginseng*, and Borneol (17). In clinical research, Compound Danshen Dripping pills and Clopidogrel Hydrogen Sulphate tablets are often combined to improve the therapeutic benefits for coronary heart disease, whose simultaneous administration significantly modifies the pharmacokinetic parameters of the compounds in *Salvia miltiorrhiza* (18). Compound Danshen tablet is widely used in the treatment of angina pectoris and coronary atherosclerosis by significantly improving microcirculation, inhibiting apoptosis, and protecting rat cardiomyocytes from MIRI through the activation of the Akt-eNOS signaling pathway (19). Guanxinning tablets play a vasodilatory role by activating the endothelial CaMKII/eNOS signaling pathway in the endothelial defect ring and calcium-related ion channels in the endothelial defect vasculature (20). Jiawei Danshen decoction has been proven to reduce the levels of myocardial enzymes creatine kinase (CK) and lactate dehydrogenase (LDH) and improve the pathological condition of myocardial tissue and the damaged myocardium (21).

## Pathophysiologic mechanisms of MIRI

**Figure 1:**
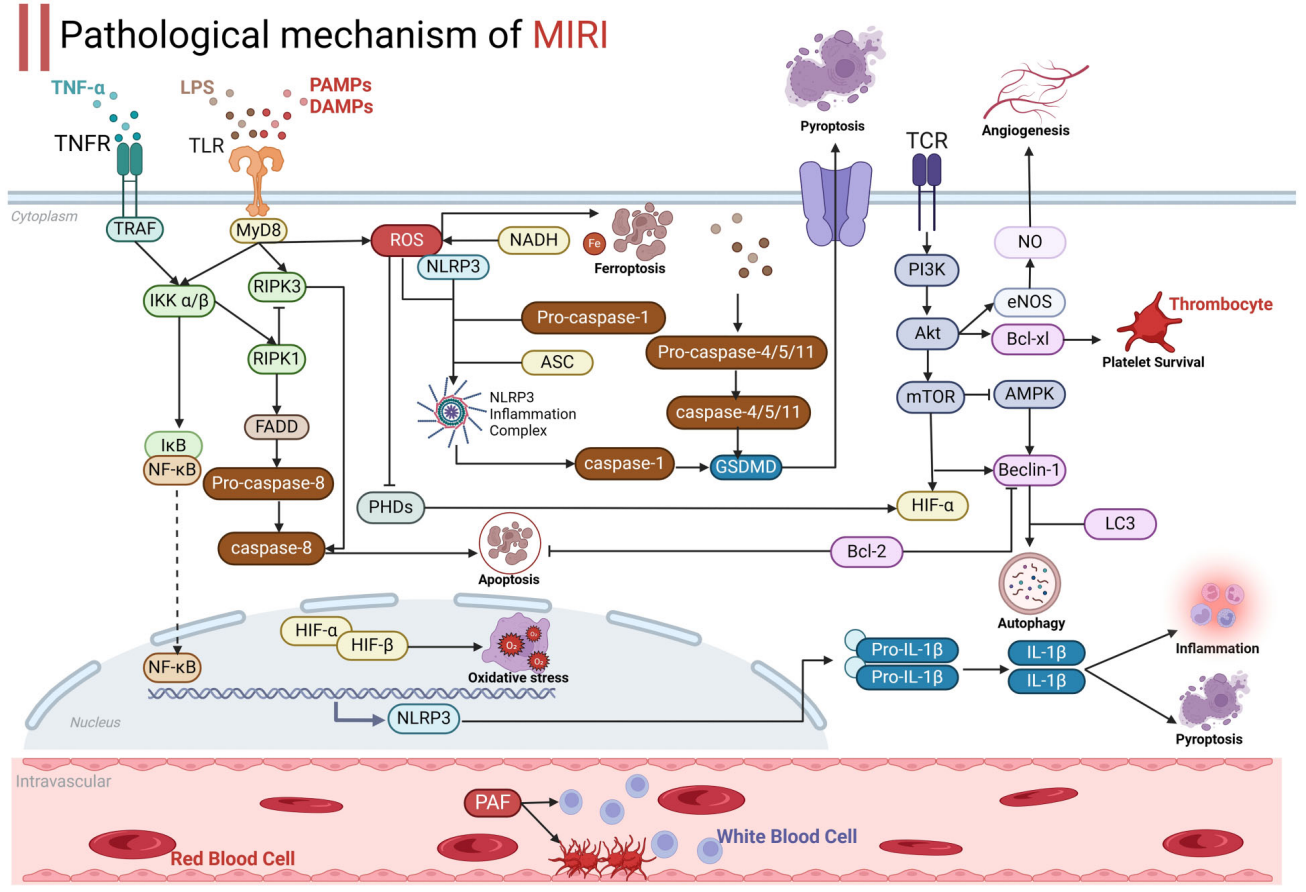
Pathological mechanism of myocardial ischemia-reperfusion injury (MIRI).

MIRI is a pathological phenomenon in which tissue damage is aggravated after myocardial ischemia is reversed following timely intravenous thrombolysis or PCI for ischemic CVDs such as MI, coronary heart disease, and HF. Cardiomyocyte dysfunction and necrosis are triggered by the cascade effect of hypoxia and energy depletion of cardiomyocytes in the ischemic period, oxidative stress, inflammatory response, and calcium overload triggered by reperfusion, and cellular injury (3). The pathogenesis of MIRI has been implicated in the release of inflammatory factors, oxidative stress, platelet aggregation, vascular endothelial dysfunction, and programmed cell death (as shown in Figure 1).

### Inflammatory response

The process of myocardial ischemia-reperfusion is accompanied by a specific type of sterile inflammation that promotes tissue infiltration and production of major inflammatory cytokines including nuclear factor-kappaB (NF-κB), tumor necrosis factor-alpha (TNF-α), high mobility group box-1 protein 1 (HMGB1), and the NOD-like receptor thermal protein domain associated protein 3 (NLRP3), among others, leading to cardiomyocyte death (22). HMGB1 is released from cardiomyocytes with MIRI and binds to toll-like receptor 2 (TLR2) and toll-like receptor 4 (TLR4), which activates NF-κB through downstream myeloid differentiation primary response 88 (MyD88) and induces cardiomyocyte apoptosis and increases myocardial infarction size through sphingosine-1-phosphate signaling on the inhibition of cAMP/Akt cascade, while causing neutrophil infiltration in the myocardium and triggering an inflammatory response that is responsible for cardiomyocyte necrosis (23). NLRP3 inflammasome, one of the key molecules of the initial inflammatory response after MIRI, induces the release of inflammatory cytokines to promote dysfunction and structural damage in the ischemic myocardium (24). Specific sterile inflammation during the process of MIRI contributes to cardiomyocyte death by facilitating the infiltration of inflammatory cells and the production of major inflammatory cytokines (such as NF-κB, TNF-α, HMGB1, and NLRP3), in which HMGB1 and NLRP3 trigger cardiomyocyte apoptosis, myocardial infarction expansion, and inflammatory response by activating associated receptors and signaling pathways.

### Oxidative stress

Oxidative stress is engaged in apoptosis and autophagy in MIRI, which is closely associated with mitochondrial oxidative dysfunction, endoplasmic reticulum dysfunction, and a decreased activity of antioxidant metabolic enzymes (25). Oxidative stress increases reactive oxygen species (ROS) production by triggering mitochondrial dysfunction, endoplasmic reticulum dysfunction, and reduced antioxidant metabolic enzyme activity, thus damaging cell membranes, proteins and nucleic acids, activating inflammatory response and apoptosis, and further aggravating cardiomyocyte injury in MIRI (26). Increased formation of xanthine oxidase, neutrophil respiratory burst, and damage to the mitochondrial electron transfer chain increase ROS generation during ischemia-reperfusion (I/R) and attack the cell membrane lipids to produce lipid peroxidation reactions, leading to destruction of cell membrane structure, increased damage to proteins and nucleic acids, activation of the inflammatory response and induction of cell apoptosis, further exacerbating oxidative stress damage to myocardial cells (17). Moreover, the mitochondrial permeability transition pore (mPTP) and the pro-apoptotic pathway are activated by ROS to initiate indirect damage, leading to tissue edema, affecting the transport of substances and signaling, etc, which leads to myocardial injury and cardiomyocyte death through a variety of mechanisms (27).

### Platelet aggregation and vascular endothelial dysfunction

In MIRI, platelets contribute to thrombosis and myocardial injury by over activating, aggregating, and releasing inflammatory mediators during the initial stage of reperfusion, while platelet-derived exosomes play an antithrombotic role by inhibiting platelet activation and alleviating atherosclerosis. The primary physiological function of platelets is to prevent atherosclerosis by repairing vascular endothelial damage induced by external stimuli or endogenous reactions through hemostasis and coagulation (28). Platelet derived exosomes (PLT-exos) are antithrombotic by inhibiting platelet activation, thereby preventing and alleviating atherosclerosis (29). Vascular inflammation is initiated by the deficiency of soluble guanylyl cyclase (sGC) in platelets, which accelerates the formation of atherosclerotic plaques (30). However, platelets are excessively activated by coagulation factors, adenosine diphosphate (ADP), and inflammatory cells to undergo adhesion and aggregation in MIRI, resulting in myocardial ischemia induced by inadequate coronary blood circulation and insufficient supply of myocardial blood and oxygen. Platelets are recruited to the infarcted area in the initial stages of reperfusion and trigger excessive aggregation through modifications in the expression of surface adhesion molecules and the release of inflammatory mediators, which leads to thrombosis due to narrowed blood vessels and reduced blood flow, thereby resulting in vessel occlusion and myocardial injury (31). The damage of cardiac microvascular endothelial cells and cardiomyocytes is a key determinant of disease progression and prognosis (32). Inflammatory response and oxidative stress are generated by MIRI, leading to dysfunction of the vascular endothelium and increased permeability of the vascular wall (33). Endothelium-derived factors comprising platelet-activating factor (PAF) and vasoconstrictors are released after endothelial cell injury that promote inflammatory responses and vasoconstriction, thus contributing to impaired vasodilatory function (34).

### Programmed cell death

#### Apoptosis

Apoptosis refers to the orderly trigger of cell death under the regulation of some genes to maintain the stability of the body's internal environment. There are three main apoptotic pathways: exogenous apoptotic pathway regulated by death receptors, endogenous apoptotic pathway regulated by cellular mitochondria, and endoplasmic reticulum stress (ERS) (35).

Apoptosis is activated in MIRI through exogenous, endogenous, and ERS pathways, in which the death receptor pathway is triggered through TNF-α and caspase-3, the mitochondrial pathway through caspase-9 and B cell lymphoma 2 (Bcl-2), and the endoplasmic reticulum pathway through ROS and calcium overload, triggering cardiomyocyte apoptosis and leading to myocardial injury (36). In the death receptor pathway, activation of caspase-3 increases the risk of death after MIRI (36). At the beginning of reperfusion, TNF-α levels are increased and death receptors are activated, promoting the transmission of death signals from the cell surface to intracellular pathways (36). There was an increase in the expression of caspase-9 in the mitochondrial pathway after MIRI, which further activated caspase-6 and caspase-3, inducing apoptosis (35). In the mitochondrial pathway, the upregulation of Bcl-2 during MIRI significantly reduced cardiomyocyte apoptosis compared to the effects of Bax overexpression in ischemic myocardial tissue (37). In the endoplasmic reticulum pathway, activation of ERS by ROS that were released by MIRI increased the activity of c-Jun N-terminal kinase (JNK), which in turn increased ROS in the mitochondria and exacerbated apoptosis (38). Conversely, excessive Ca2+ was released when ERS occurred in the endoplasmic reticulum and calcium overload was generated, which induced mitochondrial dysfunction and apoptosis (39). Signaling pathways such as adenosine monophosphate-activated protein kinase (AMPK), mitogen-activated protein kinases (MAPK), janus kinase (JAK), protein kinase C (PKC), signal transducer of activation (STAT), phosphatidylinositol-3-kinase (PI3K), caspase, fatty acid synthase/fatty acid synthase L (Fas/FasL), nuclear erythroid 2-related factor 2 (Nrf2), and extracellular regulated protein kinases (ERK) are mutually cross-influential and ultimately lead to cardiomyocyte apoptosis (40).

#### Autophagy

Cellular autophagy, also known as type II cell death, is an internal cellular self-protection mechanism and plays a dual role in MIRI. Autophagy protects cardiomyocytes and removes damaged mitochondria in the ischemic phase of MIRI, but causes autophagic death of a large number of cardiomyocytes due to excessive ROS activation and calcium overload in the perfusion phase, thereby aggravating myocardial injury (41). Dysregulation of autophagy is considered as one of the main causes of MIRI. During the ischemic phase, cardiomyocytes in the state of ischemia and hypoxia are induced to undergo autophagy by the AMPK-mechanistic target of rapamycin complex 1 (mTORC1)-UNC-51-like kinase 1 (ULK1) pathway, and its degradation products are converted into ATP to provide energy and play a protective role for cardiomyocytes damaged by ischemia and hypoxia (42). The release of apoptotic factors is also reduced by removing damaged mitochondria through autophagy. The level of autophagy in the perfusion phase is consistently increased by ROS and calcium overload signals and triggers the autophagic death of a large number of cardiomyocytes, which causes left ventricular remodeling and enlarges the infarcted area of the myocardium (43). Since cellular autophagy is fundamentally a pro-cellular survival mechanism, the degree of autophagy will determine whether it exerts a protective or detrimental effect on its own.

#### Necroptosis

Necroptosis is a novel form of cell death, which is also known as programmed necrosis. Partial cardiomyocyte necroptosis is a regulated process, mainly characterized by the rupture of the cell membrane leading to the release of intracellular products and causing a sterile inflammatory response (44). Programmed necroptosis in MIRI leads to cardiomyocyte injury and an inflammatory response through receptor-interacting protein kinase-3 (RIPK3)-mediated calcium overload, oxidative stress, and membrane rupture, while the phosphorylation of mixed lineage kinase-like (MLKL) further promotes lipid peroxidation and necrosis of the cell membrane. RIPK3, as a key protein mediating the onset of necroptosis, induces intracellular calcium ion overload, oxidative stress, and cell membrane rupture via the RIPK3-Ca2+-CaMKII-mPTP pathway, triggering cardiomyocyte injury caused by oxidative stress and mPTP opening (9). During MIRI, MLKL phosphorylation was induced by activated RIPK3 that is oligomerized on the cell membrane to mediate the lipid peroxidation of the cell membrane, leading to the rupture of the plasma membrane, releasing the damage-related molecules in the cytoplasm, triggering the inflammatory response, and inducing necroptosis (45).

#### Pyroptosis

Cellular pyroptosis is another type of cell death characterized by the swelling and rupture of cells and the release of inflammatory cytokines. The pyroptosis signaling pathway is mainly divided into the classical pathway, which is dependent on the activation of caspase-1, and the non-classical pathway, which is dependent on the activation of caspase-4/5/11 (46). Pyroptosis triggers myocardial cell membrane rupture and an inflammatory factor release in MIRI through a canonical pathway (caspase-1 and GSDMD dependent) and a non-canonical pathway (caspase-11 dependent), leading to cardiomyocyte swelling, pyroptosis and aggravation of the inflammatory response. In the canonical pathway, caspase-1 is activated by a multiprotein complex formed by the interaction of apoptosis-associated speck-like protein (ASC) and pro-caspase-1, and the cleaved gasdermin-D (GSDMD) protein causes perforation of the cell membrane and release of cell contents, which triggers an inflammatory response (47). In the non-canonical pathway, lipopolysaccharide (LPS) directly activates caspase-11 (caspase-4/5 in human origin) to cleave GSDMD and trigger pyroptosis (23). Myocardial tissue NLRP3, caspase-1, and ASC protein levels were significantly increased in the rat MIRI model, and the inflammatory response was amplified *in vivo* by increased secretion of interleukin-1beta (IL-1β), interleukin-18 (IL-18), and macrophage infiltration, leading to cardiomyocyte pyroptosis and increased severity of MIRI (48). In addition, ROS generated during I/R induce upregulation of TLR4 expression and recruit its downstream protein MyD88 to activate the NF-κB pathway to promote inflammasome formation, ultimately leading to cell swelling and pyroptosis (49).

#### Ferroptosis

Ferroptosis, a form of cell death caused by increased iron ion-dependent lipid peroxidation, is a unique phenomenon distinguished from oxidative stress. The definition of ferroptosis stems from the fact that it is effectively inhibited by lipophilic antioxidants and iron chelators, and is associated with obvious iron accumulation, but is not observed in other forms of death and is not regulated by apoptosis, pyroptosis, and autophagy inhibitors (50). Ferroptosis contributes to cardiomyocyte injury in MIRI through iron-dependent lipid peroxidation, while ferroptosis inhibitors are effective in repairing reperfusion-induced cellular injury and reduce the size of MI (51). Research has shown that the degree of MI is decreased by the application of deferoxamine (52). Additionally, ROS and iron metabolism in cardiomyocytes from transgenic mice were inhibited by the mammalian target of rapamycin (mTOR) to protect cardiomyocytes from ferroptosis and reduce cell death (50).

## *Salvia miltiorrhiza* for the treatment of MIRI

### Dashen chemical composition retrieval

In this review, we obtained 43 chemical components of *Salvia miltiorrhiza* by searching the TCMSP and PubChem databases, from which the correlation between 5 chemical elements and MIRI was verified (Supplementary Table S3).

### Literature search

Search for the chemical constituent of *Salvia miltiorrhiza* was performed with the keywords “a chemical constituent” and “myocardial ischemia reperfusion injury” or “inflammatory response” or “oxidative stress” or “platelet aggregation” or “vascular endothelial function” or “apoptosis” or “autophagy” or “necroptosis” or “pyroptosis” or “ferroptosis” in the electronic databases Pubmed, Web of Science (WOS), and the China National Knowledge Infrastructure (CNKI). The initial search was conducted on December 14, 2023, and the updated search was conducted on December 30, 2023.

Inclusion criteria were: a) studies on myocardial ischemia reperfusion injury; b) intervention studies with one of the chemical components of Danshen; c) studies conducted *in vivo* or *in vitro*; and d) experiments with no restriction on species. Exclusion criteria were: a) non-myocardial ischemia reperfusion injury and b) duplicate studies and titles or abstracts that did not meet the inclusion criteria.

## Mechanism of action of *Salvia miltiorrhiza* in the treatment of MIRI

### Inhibition of inflammatory response

Inflammation is one of the earliest responses to occur during MIRI, and remains involved throughout the whole process of myocardial injury; thus the inhibition of inflammation contributes to control MIRI. With the initiation of reperfusion therapy, the endothelial cells of cardiac vasculature begin to produce adhesion molecules, and the recruited neutrophils and monocytes secrete a large number of inflammatory cytokines, which further aggravate inflammation and myocardial injury.

**Figure 2:**
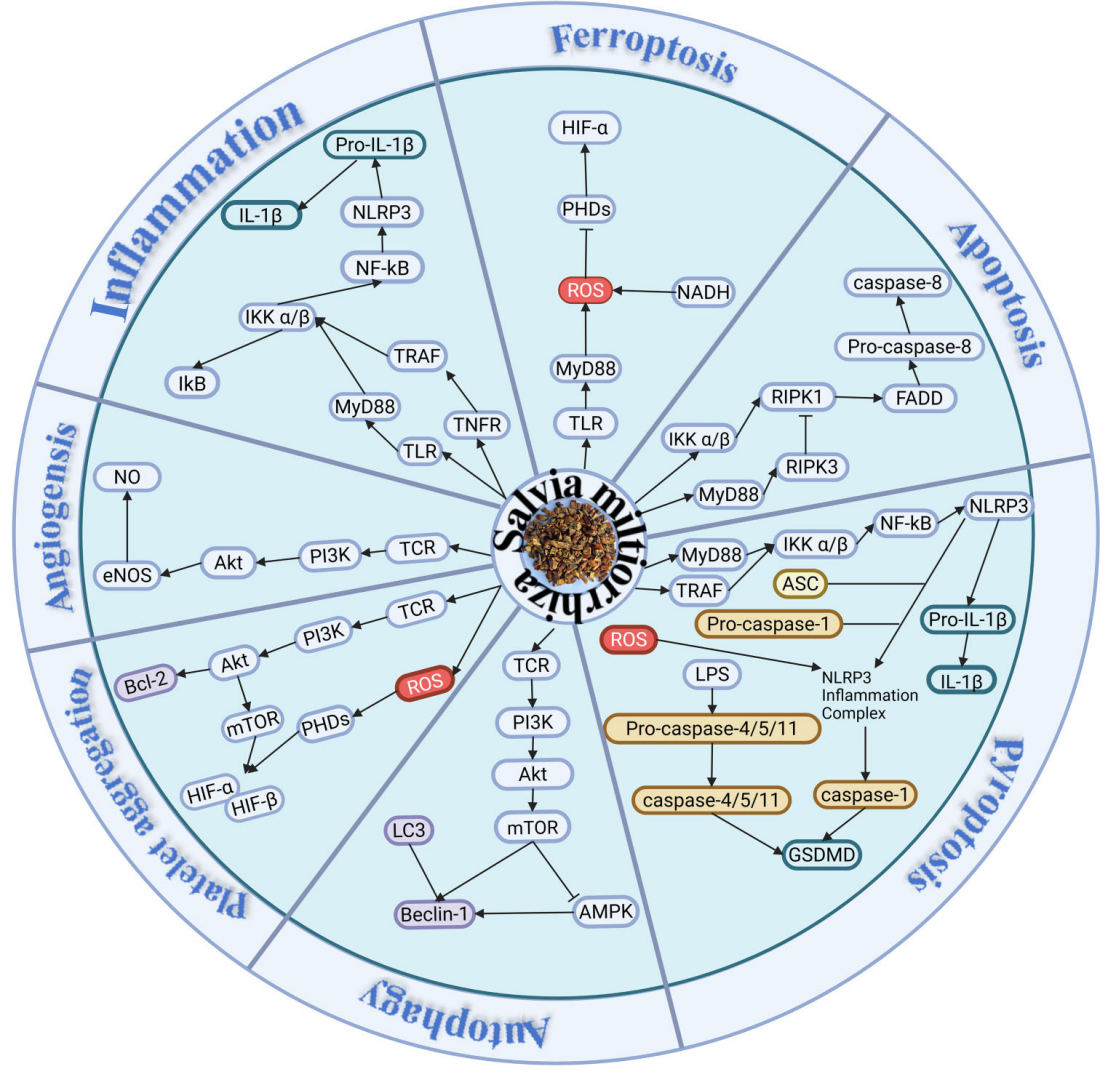
Pathophysiological mechanisms of *Salvia miltiorrhiza*.

Tanshinone IIa attenuates MIRI in rats by targeting vascular endothelial growth factor A (VEGF-A), Akt1, and methylpropyltryptamine (MPT) to modulate the PI3K/Akt signaling pathway, the hypoxia-inducible factor 1 (HIF-1) signaling pathway, and the interleukin-17 (IL-17) signaling pathway (4,53). NLRP3 inflammasomes and interleukin-6 (IL-6), as key factors of inflammatory response, are commonly investigated in the mechanistic role of *Salvia miltiorrhiza* in the treatment of MIRI (24). The activation of NLRP3 inflammasome is inhibited by tanshinone IIa, salvianolic acid B, danshensu, and rosmarinic acid (54). The level of IL-6 in myocardial tissue after MIRI is reduced under the regulation of tanshinone IIa, danshensu, and rosmarinic acid, inhibiting the release of inflammatory factors and thus alleviating MIRI (4,55). In addition, tanshinone IIa has been associated with the inhibition of HMGB1 expression and the reduction of the inflammatory response by inhibiting the increases in ROS provoked by reperfusion (25). mTOR is a key nutrient/energy sensor that combines effective nutrients with downstream metabolic processes such as protein synthesis, glycolysis, and lipid generation (53). The inflammatory response and cardiac dysfunction after MIRI are improved by salvianolic acid B through its inhibition of mTOR complex 1 (mTORC1)-dependent glycolysis and reduction of M1-polarised macrophages in MIRI hearts (56). Meanwhile, salvianolic acid B also decreases inflammatory factors such as IL-18 and caspase-1 in the cell supernatant, thereby exerting a protective effect on MIRI (57). Both danshensu and rosemarinic acid reduced the expression level of TNF-α and deregulated the NF-κB-mediated signaling pathway in myocardial tissues after MIRI, which attenuated MIRI (58). As an important protein involved in coronary artery disease caused by reduced myocardial blood supply, the protein expression of chemokine receptor 2 (CXCR2) and its downstream cyclooxygenase-2 (COX-2), intercellular adhesion molecule-1 (ICAM-1), and vascular cell adhesion molecule-1 (VCAM-1) are inhibited by danshensu, which reduces the expression level of inflammatory factors, including IL-1β, and the number of neutrophils in myocardial tissue after MIRI, thereby improving MIRI (55). Rosemarinic acid inhibits the level of inflammatory cytokine C-reactive protein (CRP), activated peroxisome proliferator-activated receptor gamma (PPARγ), and down-regulated NF-κB-mediated signaling pathway, inhibiting inflammatory responses and attenuating MIRI in rats (59). Tanshinone IIa regulates the differentiation of T helper 17 (Th17)/Treg cells, thus exerting cardioprotective effects (60). After MIRI, C-C chemokine receptor type 2+ (CCR2+)-resident macrophages are rapidly activated and participate in the subsequent inflammatory response. Studies on intramyocardial injection of tanshinone IIa combined with stem cell-derived exosomes found that miR-233-5p effectively inhibited CCR2 activation to reduce monocyte infiltration and enhanced angiogenesis, thus attenuating MIRI in rats (29). Hypoxia and reoxygenation injury to H9c2 cardiomyocytes can be ameliorated by salvianolic acid B, which targets inflammatory factors and controls the focal cell death signaling pathway (57). In addition, a new rosemarinic acid-conjugated Gd complex is available for inflammatory tissue imaging (26) (Figure 2).

### Control of oxidative stress

The aerobic metabolism of myocardial cells is abnormal due to MIRI, resulting in the production of excessive ROS that are difficult to remove promptly, which in turn react with unsaturated fatty acids on the cell membrane and damage the cell structure to exacerbate myocardial ischemia.

The levels of ROS triggered by I/R were significantly reduced by tanshinone IIa, danshensu, and rosemarinic acid, among which tanshinone IIa reduced mitochondrial damage (17). The important antioxidant enzyme heme oxygenase-1 (HO-1) participates in the corresponding mechanism of antioxidant stress through enzymatic activity and protect cardiomyocytes from damage. Sodium tanshinone IIa sulfonate, danshensu, and rosemarinic acid up-regulate the antioxidant system and regulate oxidative stress and inflammatory responses by enhancing HO-1 activity to protect cardiomyocytes and improve cardiac insufficiency (61-63). In addition, danshensu also significantly increases the activity of superoxide dismutase (SOD) and regulates the oxidative stress ability of H9c2 cardiomyocytes by increasing the level of Akt phosphorylation and the translocation of Nrf2, reducing the level of ERK phosphorylation and the levels of creatine kinase myocardial band (CK-MB) and cardiac troponin I (cTnI) in MIRI myocardial tissue (40,62). SOD, a superoxide dismutase with remarkable antioxidant activity, plays a crucial role in alleviating the severity of MIRI. Danshensu significantly increases SOD activity protecting the heart by enhancing the oxidative defense system and exerting anti-apoptotic effects (40). Malondialdehyde (MDA) is an endogenous product that reflects the degree of oxidative stress, and an increase in its level triggers an elevation of the lipid peroxidation response, which is used to assess the severity of MIRI. The levels of MDA are reduced by danshensu and salvianolic acid A, which inhibit myocardial lipid peroxidation and protect the vascular endothelium and myocardial tissue (37,63). Salvianolic acid A exerted a protective effect on AC16 cardiomyocytes by attenuating oxidative stress through regulating sirtuin 1 (SIRT1) and increasing plasma NO levels (64). Additionally, oxidative stress was inhibited by salvianolic acid B to protect cardiac cells from I/R injury through modulation of the tripartite motif (TRIM) proteins in the TRIM8/glutathione peroxidase 1 (GPX1) axis (65) (Figure 2).

### Antiplatelet aggregation and modulation of vascular endothelial function

Platelets are overactivated by coagulation factors, ADP, and inflammatory cells after contact with subendothelial collagen. Vascular stenosis and reduced blood flow are triggered by the physiopathological mechanisms that occur after reperfusion, ultimately resulting in vascular obstruction and myocardial injury (28). A large number of platelets and neutrophils are activated during I/R and adhere to the inner surface of cardiac capillaries leading to damage to the endothelial cells and peripheral tissues, blockage of the capillaries, and the formation of microthrombi to impede blood flow (32).

Once platelets are activated during I/R, p-selectin present on activated platelets is cleaved from the cell membrane to form a soluble form that is detected in plasma (31). Salvianolic acid A-pretreated reperfusion rats had significantly reduced levels of serum p-selectin, TNF-α, IL-1β, NO, cardiac troponin T (cTnT), and CK-MB and inhibited platelet activation and aggregation as well as inflammation, resulting in improved coronary blood flow (66). Danshensu also exerted a cardioprotective effect against MIRI in rats through antiplatelet aggregation (67). In addition, a new acetylated danshensu and tetramethylpyrazine conjugate (ADTM) has been demonstrated to possess cardioprotective effects such as antioxidant, modulation of vasodilatation, pro-angiogenesis, and antiplatelet aggregation (68). Platelet-activating factor receptor (PAFR) signaling was downregulated by ADTM, and apoptosis and inflammatory response of cardiomyocytes during hypersensitive response (HR) were inhibited by siRNA to protect MIRI (34). Studies have shown that rosemarinic acid inhibits platelet aggregation and regulates its function by inhibiting protein tyrosine phosphorylation. Release of platelet microvesicles is significantly reduced by activating the Kelch-like ECH-associated protein 1 (Keap1)-Nrf2-Are antioxidant system to inhibit platelet-derived growth factor (PDGF)-BB-induced proliferation and migration of vascular smooth muscle cells (VSMCs) (69). Vascular endothelial growth factor (VEGF) plays an important role in promoting angiogenesis and vascular repair of injured and ischemic tissues (33). Cardiac function after MI was improved by the miR-499-5p/phosphatase and tensin homolog (PTEN) signaling pathway through the downregulation of miRNA-499 expression by tanshinone IIa, which induced angiogenesis (70). The increase in the formation and expression of VEGF, vascular endothelial growth factor receptor-2 (VEGFR-2), and matrix metalloproteinase 9 (MMP-9), as well as the number and function of endothelial progenitor cells (EPCs), promoted the enhancing effect of salvianolic acid A on ischemia-induced neovascularization one week after MI surgery (71) (Figure 2).

### Inhibition of programmed cell death

#### Apoptosis

In MIRI, apoptosis is exacerbated by the overload of Ca2+, the increase of ROS, and the disorder of energy metabolism. The mechanism of apoptosis begins with the release of proteins including cytochrome C (CytC) from mitochondria, which interact with the protein Apaf1 within the cytoplasm to form complexes and activate caspase-9 protease and subsequently caspase-3 and caspase-6 elevate the irreversible proteases activity, causing the cell to enter an apoptotic state (37).

Mammalian transient receptor potential (TRP) channels serve as a new starting point for studying the molecular basis of Ca2+ entry. It has been shown that levels of transient receptor potential-6 (TRPC6) protein, Ca2+, and apoptosis were reduced by danshensu pretreatment in I/R injury-induced H9c2 cells (39). ROS, as one of the major triggers of mPTP opening, is capable of preventing a decrease in mitochondrial ATP production by increasing the stability of its membrane potential. MIRI was remediated by danshensu through downregulation of forkhead box protein O1 (FoxO1) mRNA expression, reduction of ROS production, inhibition of excessive mPTP release, and stabilization of membrane potential (72). Tanshinone IIa increased MMP and Bcl-2/Bax ratio, activated caspase-3, and shut down mPTP to reduce the release of CytC to alleviate myocardial cell apoptosis and achieve myocardial protection (37). In addition, tanshinone IIa decreased the phosphorylation levels of p38MAPK and ERK and significantly increased the phosphorylation level of Akt in H9c2 cells (40). The glutathione peroxidase 4 (GPX4) and JNK apoptotic signaling pathways are degraded by the natural antioxidant salvianolic acid B to alleviate ROS-related tissue damage, downregulate JNK phosphorylation, and caspase-3 expression to inhibit cell apoptosis (38). Rosmarinic acid exerted an anti-apoptotic effect on cardiomyocytes through cardiac fibroblasts by inhibiting the activation of activated nuclear factor of activated T cells (NFAT) and the expression of matrix metalloproteinase 7 (MMP-7) (73) (Figure 2).

#### Autophagy

Cellular autophagy is a programmed and genetically controlled form of natural cell death. Cellular autophagy promotes cell survival during the myocardial ischemia phase and cell death during the reperfusion phase. Ischemia stimulates autophagy through an AMPK/mTOR target protein-dependent mechanism, while reperfusion injury will stimulate autophagy through a mechanism dependent on the key autophagy molecule yeast autophagy-related gene (*ATG6*) homolog (Beclin-1), but independent of AMPK, thus playing a dual role (41).

As a novel danshensu/ligustrazine derivative, DT-010 is a potential candidate for the treatment of MIRI, which reduces the phosphorylation of 5'-AMPK through the MAPK-mTOR-Ulk1 signaling pathway, promotes the phosphorylation of mTOR and unc-51-like Ulk1, upregulates the protein expression of peroxisome proliferator-activated receptor-gamma coactivator-1alpha (PGC-1α), Nrf2, Tfam, and HO-1, and triggers Nrf2 nuclear translocation, thereby inhibiting autophagy in H9c2 cells (74). Tanshinone IIa activates the AMPK-mTOR signaling pathway by up-regulating AMPK and down-regulating mTOR, thereby inhibiting apoptosis and inducing autophagy to protect cardiomyocytes and improve cardiac function (42). In addition, some studies have shown through *in vitro* and *in vivo* experiments that tanshinone IIa is able to regulate the AMPK-mTOR signaling pathway through the miR-241-3p/ATG 16L1 axis (41). Moreover, tanshinone IIa was shown to attenuate the up-regulation of autophagy-related proteins light chain 3 (LC3)-II, Beclin-1, and Sirt6 in the ischemic model of tanshinone IIa intervention (43). In addition, during the ischemic phase, tanshinone IIa could also activate kruppel-like factor 4 (KLF4) by inhibiting miR-375 to enhance macrophage autophagy and provide energy to damaged cardiomyocytes (75) (Figure 2).

#### Necroptosis

The necroptosis mechanism is mainly through the phosphorylation of MLKL by necrosomes formed by the interaction of receptor-interacting protein 1 (RIP1) and receptor-interacting protein 3 (RIP3), which leads to mitochondrial dysfunction and the release of damage-associated molecular pattern (DAMP) through mPTP, leading to an inflammatory response that triggers necroptosis (45). Necroptosis was stimulated with tanshinone IIa by the impulse of caspase inhibitors (9). Rosmarinic acid induces apoptosis and necrosis in a ROS-independent DNA damage and caspase-independent manner (44) (Figure 2).

#### Pyroptosis

Pyroptosis includes two signaling pathways, the canonical pathway (dependent on caspase-1 activation) and the non-canonical pathway (dependent on caspase-4/5/11 activation). Inhibition of NLRP3 and caspase-1 activity reduces the inflammatory response and cardiomyocyte pyroptosis, which improves MIRI (48). In addition, the reactive oxygen species-induced TLR4/NF-κB signaling pathway was also involved in the formation of inflammatory vesicles and cellular pyroptosis (49).

Tanshinone IIa attenuates myocardial injury by inhibiting the TLR/NF-κB p65 signaling pathway and the TLR4/MyD88/NF-κB/NLRP3 cascade, thereby inhibiting cardiomyocyte apoptosis (49). Tanshinone IIa sulfonate regulates endothelial cell mitochondrial function and focal death via the AMPK-dependent mitochondrial pathway (76). Salvianolic acid B is able to promote the accumulation of Nrf2 in the nucleus, activate the Nrf2/NLRP3 signaling pathway, regulate the NF-κB signaling pathway. The AMPK/Fox04/kruppel-like factor 2 (KLF2) and syndecan-4/receive array coil 1 (Rac 1)/activating transcription factor 2 (ATF 2) signaling pathways were used to reduce the level of intracellular ROS and reverse the significantly upregulated NLRP3, caspase-1, GSDMD, and IL-1β, and inhibit pyroptosis effectively preventing damage caused by endoplasmic reticulum stress, thereby alleviating I/R injury (36,46,75). Salvianolic acid A and danshensu significantly inhibited NLRP3-dependent inflammatory vesicle activation and inhibited the conversion of downstream proteins including IL-1β, IL-18, and GSDMD-N from inactive cytoplasmic precursors to mature active forms, thereby inhibiting cellular focal death (47) (Figure 2).

#### Ferroptosis

Tanshinone IIa pretreatment significantly inhibited the effect of erastin on H9c2 cells and activated Nrf2 in human coronary artery endothelial cells, thereby inhibiting ferroptosis (37,77 [Bibr B78] [Bibr B79] [Bibr B80] [Bibr B81] [Bibr B82] [Bibr B83] [Bibr B84] [Bibr B85] [Bibr B86] [Bibr B87] [Bibr B88] [Bibr B89] [Bibr B90]). Meanwhile, salvianolic acid B also exerted cardioprotective effects by activating the Nrf2 signaling pathway, regulating ROS, and inhibiting ferroptosis (38). Voltage-dependent anion channel 1(VDAC1) is a key component involved in metabolites, nucleotides, and ion transport on the outer mitochondrial membrane, and its up-regulation activates ferroptosis in I/R, which was averted by tanshinone IIA (37). GPX4 reduces hydrogen peroxide and is a vital negative regulator of ferroptosis whose inactivation directly induces ferroptosis. Salvianolic acid B inhibits ferroptosis in cardiomyocytes during MIRI by reducing ubiquitin-proteasome degradation of the GPX4 pathway, a marker gene for ferroptosis, and by revealing a GPX4/ROS/JNK-mediated crosstalk mechanism (38). In addition, tanshinone IIa could ameliorate ferroptosis through a PI3K/Akt/mTOR-mediated anti-ferroptosis mechanism, thereby significantly inhibiting I/R-induced GPX4 levels (37) (Figure 2).

## Discussion

The death of cardiomyocytes induced by MIRI treatment mainly includes reperfusion-induced arrhythmia, myocardial stunning, microvascular obstruction, and lethal MIRI. Myocardial reperfusion therapy has made some progress with the continuous development of PCI techniques and antithrombotic drugs that keep the infarct-related coronary arteries patent. However, the drugs used to modulate the reperfusion injury and activate the kinase survival signaling pathway to maintain mitochondrial function after acute MIRI are hepatotoxic. Therefore it is worthwhile to search for effective and safe targeted drugs. Traditional Chinese medicine (TCM) offers a potential solution for the control and treatment of MIRI due to its multi-pathway and multi-target characteristics.

*Salvia miltiorrhiza* is a traditional Chinese herb widely used in cardiovascular diseases with pharmacological effects, such as inhibition of inflammatory responses, attenuation of oxidative stress, improvement of platelet aggregation and vascular endothelial dysfunction, and programmed cell death. In this review, five chemical components related to MIRI were retrieved based on the current research on *Salvia miltiorrhiza* and its derived natural compounds. Inflammatory reaction, oxidative stress, platelet aggregation, vascular endothelial function, apoptosis, autophagy, necroptosis, pyroptosis, ferroptosis, and other aspects are considered as the potential protective mechanisms of the five chemical components of MIRI. Antioxidative stress is involved in the five chemical components, and inflammatory response and pyroptosis are involved in four components. *Salvia miltiorrhiza* mainly plays the role in antioxidative stress, anti-inflammatory response, and inhibition of pyroptosis in the treatment of MIRI.

Some limitations in the existing studies should be mentioned: a) the studies are mostly *in vivo* and *in vitro*, and there is still a lack of high-quality and large-scale clinical experiments with double-blind, multicenter, and randomized design; b) the pathological mechanisms of MIRI and the pharmacological mechanisms of action of *Salvia miltiorrhiza* and its active ingredients in MIRI were not well-known in depth. In addition, the studies on the effects of water-soluble and fat-soluble components of *Salvia miltiorrhiza* on MIRI under different pathological stages and intervention mechanisms lack consistency, certainty, and stability, whereas synergism and interactions between different active components are not fully clarified; c) the quality of *Salvia miltiorrhiza* for clinical use is still not standardized worldwide. Due to the considerable differences in the medicinal herbs cultivated in different regions, the pharmacological basis of the substances present in these herbs has not been clearly defined.

Despite these limitations, natural compounds with multi-target actions, such as *Salvia miltiorrhiza*, have gained attention as potential alternatives. To overcome these limitations, further analysis is needed to explore the mechanism and targets of *Salvia miltiorrhiza* in the treatment of MIRI, including: a) double-blind, multi-center, and randomized controlled studies in high-quality, large-scale clinical trials to further clarify the clinical feasibility of *Salvia miltiorrhiza* compound preparation for the treatment of MIRI; b) investigate the pathological mechanisms associated with MIRI and the pharmacological mechanisms by which *Salvia miltiorrhiza* and its active ingredients act on MIRI to improve the stability and safety of the pharmacological effects. More components with medicinal value should be extracted from the active ingredients of *Salvia miltiorrhiza* to optimize the pharmacological effect and provide preparations with superior efficacy and fewer adverse reactions for the improvement of MIRI; c) high-performance liquid chromatography (HPLC) and mass spectrometry should be used to explore the active ingredients of *Salvia miltiorrhiza* and standardized the use of *Salvia miltiorrhiza* globally.

## Conclusion

This review examines the four pathophysiological mechanisms of *Salvia miltiorrhiza* and its active components in mitigating inflammation, oxidative stress, platelet aggregation, vascular endothelial function, and programmed cell death during MIRI. Although *Salvia miltiorrhiza* has been widely used in the treatment of cardiovascular diseases, the specific mechanisms by which it improves MIRI have not been fully elucidated. Further understanding of the protective benefits of *Salvia miltiorrhiza* on the myocardium will contribute to the development of new therapeutic approaches for MIRI.

## Supplementary Material

Click here to view [pdf].
