# Supplementary material for: Salvia miltiorrhiza: insights on the protective effect and mechanism of myocardial ischemia-reperfusion injury
Source: Braz J Med Biol Res. 2025 Oct 6;58:e14723. doi: 10.1590/1414-431X2025e14723 (PMC12513696; doi:10.1590/1414-431X2025e14723)
Supplement: Supplementary file 1 [file 1414-431X-bjmbr-58-e14723-suppl.pdf]

Table S1. Key compounds of *Salvia miltiorrhiza*.

Molecule name	Structure	Molecular Formula	Molecular Weight	Isomeric/Canonical SMILES
Phenolic acids				
Salvianolic acid A	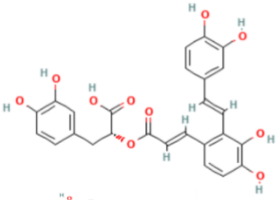	$C_{26}H_{22}O_{10}$	494.4 g/mol	C1=CC(=C(C=C1C[C@H](C(=O)O)OC(=O)/C=C/C2=C(C(=C(C=C2)O)O)/C=C/C3=CC(=C(C=C3)O)O)O)O
Salvianolic acid B	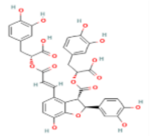	$C_{36}H_{30}O_{16}$	718.6 g/mol	C1=CC(=C(C=C1C[C@H](C(=O)O)OC(=O)/C=C/C2=C3[C@H]([C@H](OC3=C(C=C2)O)C4=CC(=C(C=C4)O)O)C(=O)O[C@H](CC5=CC(=C(C=C5)O)O)C(=O)O)O)O
Diterpenoids				
Tanshinone I	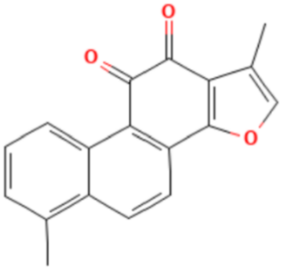	$C_{18}H_{12}O_3$	276.3 g/mol	CC1=C2C=CC3=C(C2=CC=C1)C(=O)C(=O)C4=C3OC=C4CO
Tanshinone IIa	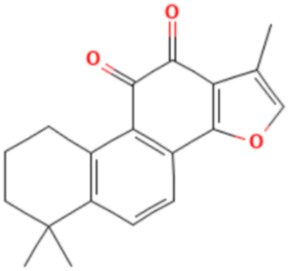	$C_{19}H_{18}O_3$	294.3 g/mol	CC1=COC2=C1C(=O)C(=O)C3=C2C=CC4=C3CCCC4(C)C
Tanshinone IIb	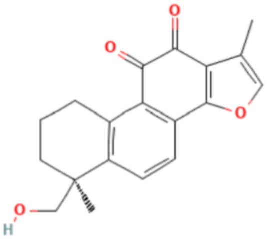	$C_{19}H_{18}O_4$	310.3 g/mol	CC1=COC2=C1C(=O)C(=O)C3=C2C=CC4=C3CCC[C@]4(C)CO
Miltirone	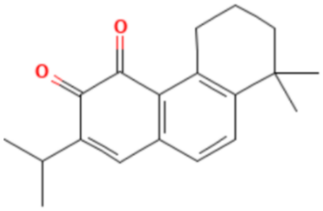	$C_{19}H_{22}O_2$	282.4 g/mol	CC(C)C1=CC2=C(C3=C(C=C2)C(CCC3)(C)C)C(=O)C1=O

Tanshinol B	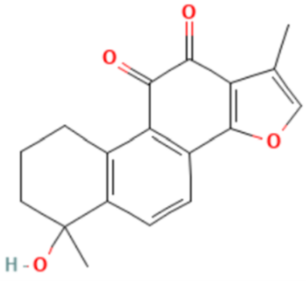	$C_{18}H_{16}O_4$	296.3 g/mol	CC1=COC2=C1C(=O)C(=O)C3=C2C=CC4=C3CCCC4(C)O
Cryptotans-hinone	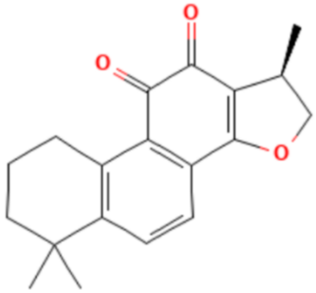	$C_{19}H_{20}O_3$	296.4 g/mol	C[C@H]1COC2=C1C(=O)C(=O)C3=C2C=CC4=C3CCCC4(C)C
Sclareol	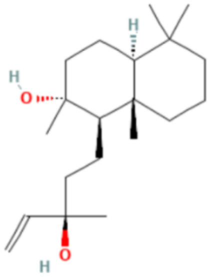	$C_{20}H_{36}O_2$	308.5 g/mol	C[C@]12CCCC([C@@H]1CC[C@@]([C@H]2CC[C@](C)(C=C)O)(C)O)(C)C
Triperpenoids α-amyrin	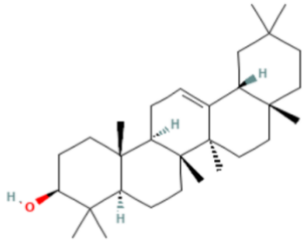	$C_{30}H_{5}O_{0}$	426.7 g/mol	C[C@@H]1CC[C@@]2(CC[C@@]3(C(=CC[C@H]4[C@]3(CC[C@@H]5[C@@]4(CC[C@H](C5(C)C)O)C)C)[C@@H]2[C@H]1C)C)C
Flavonoid Luteolin	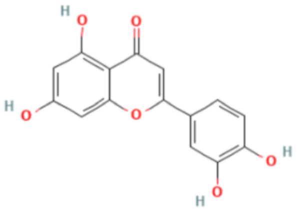	$C_{15}H_{10}O_6$	286.24 g/mol	C1=CC(=C(C=C1C2=CC(=O)C3=C(C=C(C=C3O2)O)O)O)O

SMILES: Simplified Molecular Input Line Entry System.

Table S2. *Salvia miltiorrhiza*-related proprietary Chinese medicines.

Form	Drug name	Components	Type of study	Experimental model	Administration method	Trend of key indicators or targets of research	Efficacy	References
Tablet	Danshen Tablet	*Salvia miltiorrhiza*	*In vivo*	Rats with reperfusion after ligation of left anterior descending coronary artery	Gavage	Caspase-3↓, CK↓, LDH↓, cTnI↓, p-Akt↑, p-eNOS↑, bcl-2↑, NO↑	Protected cardiomyocytes against MI/R injury and inhibits apoptosis	(78)
	Compound Danshen Tablet	*Salvia miltiorrhiza*, *Panax notoginseng*, Borneol	*In vivo*	Isoproterenol-induced myocardial injury in high-fat-diet fed mice	Gavage	α-SMA↓, TGF-β↓	Limited cardiac fibrosis	(19)
	Xinkeshu Tablet	*Salvia miltiorrhiza*, Radix Puerariae, *Panax notoginseng*, Hawthorn, Costustoot	*In vitro*	Zebrafish embryos and human umbilical vein endothelial cells	Medicated serum	Akt↑, PI3K↑, kdr↑, flt-1↑	Promoted angiogenesis	(79)
	Guanxinning Tablet	*Salvia miltiorrhiza*, Chuanxiong Rhizoma	*In vitro*	Rabbits with Isolated thoracic aortic rings	Incubation	NO↑	Exerts Endothelium-Mediated Vasodilation	(20)
	Xueshuan Xinmaining Tablet	*Salvia miltiorrhiza*, Chuanxiong Rhizoma, *Sophora japonica*, Leech, Ilex Pubescens, Bovis Calculus, Synthetic musk, Ginseng Folium, Borneol, Bufonis Venenum	*In vivo*	Rats with epinephrine	Gavage	Arachidonic acid↓, leukotriene A4↓, 5-HETE↓	Ameliorate blood stasis syndrome	(80)
	Danlou Tablet	*Salvia miltiorrhiza*, Trichosanthis peel, *Allium macrostemon*, Radix Puerariae, Chuanxiong Rhizoma, Red paeony root, Alismatis Rhizoma, *Astragalus membranaceus*, Drynariae Rhizoma, Curcumae Radix	*In vitro*	Human AC16 cells and neonatal rat cardiomyocytes	Medicated serum	Bax/Bcl2↓, Caspase-3↓, BIM↓, PUMA↓	Protect against cardiac remodeling and dysfunction after myocardial I/RI	(81)
Drop Pill	Compound Danshen Dripping Pill	*Salvia miltiorrhiza*, *Panax notoginseng*, Borneol	*In vivo*	Dual deficient mice induced by hypercholesterolemia/atherosclerosis in apolipoprotein E (ApoE) and LDL receptor (LDLR)	Gavage	Wnt↓, KDM4A↓	Anti-myocardial dysfunction and anti-fibrosis	(17)
	Qishen Yiqi Dripping Pill	*Astragalus membranaceus*, *Salvia miltiorrhiza*, *Panax notoginseng*, Rosewood	*In vivo*	Rats with reperfusion after ligation of left anterior descending coronary artery	Gavage	P-PI3K↑, p-Akt↑, p-mTOR↑, ATG5↓, Beclin1↓, p53,LC3BI/II↓, Bcl-2↑, NLRP3↓, ASC↓	Ameliorated myocardial I/R injury	(54)
	Shexiang	Synthetic musk, *Salvia*	*In vivo*	Rats with ligation of left	Gavage	Dectin-1↓, TNF-α↓	Alleviated M1	(82)

	Tongxin Dropping Pill	*miltiorrhiza*, Ginseng Folium, Bufonis Venenum, Bovis Calculus, Xiongdan(Fel Ursi) powder, Borneol		anterior descending coronary artery			macrophage polarization - induced inflammation and endothelial dysfunction, against coronary microvascular dysfunction	
Capsule	Guanxin Shutong Capsule	Choerospondiatis Fructus, *Salvia miltiorrhiza*, Caryophylliflos, Borneol, Bamusaeconcretiosilicea	*In vivo*	Rats with ligation of left anterior descending coronary artery	Gavage	CK↓, LDH↓, AST↓	Anti-coronary heart disease	(83)
	Yixinshu Capsule	Ginseng, Ophiopogonis Radix, *Astragalus membranaceus*, Schisandra chinensis, *Salvia miltiorrhiza*, Chuanxiong Rhizoma, Hawthorn	*In vivo*	Rat model of cardiac hypertrophy induced by abdominal aortic constriction	Gavage	ANP↓, BNP↓, cTnI↓	Attenuate cardiac hypertrophy	(84)
	Tengdan Capsule	Gambir plant nod, *Salvia miltiorrhiza*, Prunellae Spica, Porcine Bile Paste, Herba Taxilli, Plantaginis Semen, Chuanxiong Rhizoma, *Panax notoginseng*, Stephaniae Tetrandrae Radix, *Astragalus membranaceus*	*In vivo*	Adult spontaneously hypertensive rats	Gavage	TGF-β↓	Lowered blood pressures	(85)
	Dan Deng Tong Nao Capsule	*Salvia miltiorrhiza*, Erigeron Breviscapus, Chuanxiong Rhizoma, Radix Puerariae	*In vivo*<br>*In vitro*	Rats with middle cerebral artery occlusion/reperfusion<br>Brain microvascular endothelial cells with oxygen-glucose deprivation/reoxygenation	Gavage<br>Medicated serum	HIF-1α↑, CD31↑, CD34↑, VEGFR2↑, VEGFRA↑, BFGF↑, BDNF↑	Promoted angiogenesis of cerebral microvasculature, protect cerebral ischemia reperfusion injury	(86)
	Qili Qiangxin Capsule	*Astragalus membranaceus*, Lepidii semen, *Salvia miltiorrhiza*, Ginseng, Heishun Tablets, Alismatis Rhizoma, Polygonati Odorati Rhizoma, Ramulus Cinnamomi, Safflower, Periplocae Cortex, Orange peel	*In vivo*	Rats with ligated left anterior descending coronary artery	Gavage	TGF-β1↓, p-Smad3↓, p-Smad7↑, Col- I↓, a-SMA↓, TNF-α↓, IL-6↓, NF-κB↓, p-IκBα↓	Protective effects against cardiac remodeling, reducing myocardial inflammation and fibrosis	(87)

Naoxintong Capsule	Synthetic musk, *Salvia miltiorrhiza*, Red paeony root, Angelicae Sinensis Radix, Persicae Semen, Safflower, Olibanun, Myrrh, Spatholobus Suberectus Dunn, Radix Achyranthis Bidentatae, Ramulus Cinnamomi, Ramulus Mori, Pheretima, Scorpio, Leech	*In vivo*	Rats with ligated left anterior descending coronary artery	Gavage	LDH↓, CK↓, CK-MB↓, CD45↓	Decreased infarct size, inhibited platelets activation, inhibited infarct expansion	(88)

Table S3. Mechanism of action of *Salvia miltiorrhiza* chemical constituents in relation to myocardial ischemia-reperfusion injury (MIRI).

Mechanism		Compound				
		Tanshinone IIa	Salvianolic acid B	Salvianolic acid A	Danshensu	Rosmarinic acid
Inflammatory response	Type of study	*In vivo*	*In vitro*	–	*In vivo*	*In vivo*
	Experimental model	Rats with reperfusion after ligation of left anterior descending coronary artery	Bone marrow-derived macrophages		Rats with reperfusion after ligation of left anterior descending coronary artery	Rats with reperfusion after ligation of left anterior descending coronary artery
	Administration method	Intravenous injection	Medicated serum		Tail vein injection	Tail vein injection
	Trend of key indicators or target of research	PI3K↑, p-Akt↑, Akt↑, p-mTOR↑, mTOR↑, p-eNOS↑, eNOS↑	IL-6↓, iNOS↓, CCL2↓, TNF-α↓, Arg1↑, Clec10a↑, Mrc↑		CXCR1↓, NF-κB↓, COX-2↓, ICAM-1↓, VCAM-1↓	CXCR1↓, NF-κB↓, COX-2↓, ICAM-1↓, VCAM-1↓
	Reference	(53)	(56)		(55)	(55)
Oxidative stress	Type of study	*In vivo*	*In vitro*	*In vivo*<br>*In vitro*	*In vitro*	*In vitro*
	Experimental model	Rats with reperfusion after ligation of left anterior descending coronary artery	AC16 cardiomyocytes with I/R	mice<br>AML-12 (alpha mouse liver 12) cell	H9c2 cell with H_2O_2injury	Human cardiomyocyte cell line (AC16) and human induced pluripotent stem-cell-derived cardiomyocytes (hiPSC-CMs)
	Administration method	Intravenous injection	Medicated serum	Oral administration<br>Medicated serum	Medicated serum	Medicated serum
	Trend of key indicators or target of research	TNF-α↓, HO-1↑, NF-κB↓	TRIM8↓, GPX1↓	GSH↑, SIRT1↑	SOD↓, MDA↓, ERK↓, AKT↑, p38 MAPK↓	Caspase-9↓, ROS↓, HO-1↑,IL-6↓, pro-BNP↓, cTnI↑
	Reference	(89)	(65)	(64)	(40)	(61)
Platelet aggregation	Type of study	–	–	*In vivo*	*In vivo*<br>*In vitro*	*In vivo*<br>*In vitro*
	Experimental model			Rats with reperfusion after ligation of left anterior descending coronary artery	Rats with reperfusion after ligation of left anterior descending coronary artery<br>Neonatal rat ventricular myocytes with hypoxia re-oxygenatcion injury	Rats with ligation of left anterior descending coronary artery<br>Human aortic VSMCs
	Administration method			Intravenous injection	Intravenous injection<br>Medicated serum	Gavage<br>Medicated serum

	Trend of key indicators or target of research			cTnT↓, CK-MB↓, IL-1β↓, TNF-α↓,NO↓	Caspase-3↓, TNF-α↓, IL-1β↓, PAFR↓	Keap1, HO-1↑, NQO1↑, GCLM↑, GST↑, Nrf2↑, NF-κB↑
	Reference			(66)	(68)	(69)
Vascular endothelial function	Type of study	*In vivo*	–	*In vivo*	–	–
	Experimental model	Rats with ligation of left anterior descending coronary artery		Rats with reperfusion after ligation of left anterior descending coronary artery		
	Administration method	Intravenous injection		Intravenous injection		
	Trend of key indicators or target of research	miR-499↓, PTEN↑		cTnT↓, CK-MB↓, IL-1β↓, TNF-α↓,NO↓		
	Reference	(70)		(66)		
Apoptosis	Type of study	*In vitro*	*In vivo*<br>*In vitro*	–	*In vitro*	*In vivo*<br>*In vitro*
	Experimental model	H9c2 cells with anoxia/reoxygenation (A/R) injury	Rats with reperfusion after ligation of left anterior descending coronary artery<br>H9c2 cardiomyocyte with hypoxia/reoxygenation damage		H9c2 cardiomyocyte with hypoxia/reoxygenation damage	Mice with single intraperitoneal injection of DOX<br>Neonatal rat cardiomyocytes and cardiac fibroblasts
	Administration method	Medicated serum	Administered intraperitoneally<br>Medicated serum		Medicated serum	Oral administration<br>Medicated serum
	Trend of key indicators or target of research	VDAC1↓, MMP↑, Bcl-2/Bax↑, Caspase-3↑	GPX4↑, JNK↓, Bax↓, Bcl-2↑, Caspase-3↓,p-MAPK↓		JNK↓, p-JNK↓, NF-κB↓, TRPC6↓	NFAT↓, MMP7↓, Fas L↓
	Reference	(37)	(38)		(39)	(73)
Autophagy	Type of study	*In vivo*<br>*In vitro*	–	–	*In vivo*<br>*In vitro*	–
	Experimental model	Rats with left anterior descending ligation<br>H9C2 cell with hydrogen peroxide			Rats with left anterior descending ligation<br>H9C2 cell with t-BHP	
	Administration method	Gavage<br>Medicated serum			Intravenous injection<br>Medicated serum	
	Trend of key indicators or target of research	LC3↑, Beclin1↑, ATG3↑, ATG7↑, p62↓			Bax↓, Bcl-2↑, Caspase-3↓, Cyt C↓	
	Reference	(42)			(74, 90)	
Necroptosis	Type of study	*In vitro*	–	–	–	*In vitro*
	Experimental model	Human hepatocellular carcinoma HepG2 cells				Human umbilical vein endothelial cells
	Administration method	Medicated serum				Medicated serum
	Trend of key indicators or target of research	RIP1↓, RIP3↓, Caspase-8↓				Caspase-1↓, NLRP3↓, LDH↓, IL-1β↓
	Reference	(9)				(44)
Pyroptosis	Type of study	*In vivo*<br>*In vitro*	*In vitro*	*In vivo*	*In vitro*	-
	Experimental model	Rats with left anterior descending ligation<br>H9C2 cardiomyocytes with hypoxia-reoxygenation	Bone marrow specimens	Diabetic ApoE-/-mice with streptozotocin	Cerebral microvascular endothelial cells with OGD/R	
	Administration method	Oral administration<br>Medicated serum	Medicated serum	Intraperitoneal injection	Medicated serum	
	Trend of key indicators or target of research	NT-pro-BNP↓, IL-1β↓, IL-18↓, TLR4↓, NF-κB p65↓, NLRP3↓, Caspase-1↓,	ROS↓, HO-1↑, SOD2↑, TXNIP↓, NLRP3↓, KLF2↑, p-FoxO4↓	NLRP3↓, ASC↓, GSDMD↓, Caspase-1↓	NLRP3↓, CLIC4↓	

		GSDMD-N↓				
	Reference	(49)	(75)	(8)	(47)	
Ferroptosis	Type of study	*In vitro*	*In vivo*<br>*In vitro*	–	–	–
	Experimental model	Human coronary artery endothelial cells	Rats with reperfusion after ligation of left anterior descending coronary artery<br>H9c2 cardiomyocyte with hypoxia/reoxygenation damage			
	Administration method	Medicated serum	Administered intraperitoneally<br>Medicated serum			
	Trend of key indicators or target of research	Nrf2↑	GPX4↑, JNK↓, Bax↓, Bcl-2↑, Caspase-3↓,p-MAPK↓			
	Reference	(77)	(38)			
